# Pathological Defects in a Drosophila Model of Alzheimer’s Disease and Beneficial Effects of the Natural Product Lisosan G

**DOI:** 10.3390/biom14070855

**Published:** 2024-07-15

**Authors:** Silvia Bongiorni, Elisabetta Catalani, Ivan Arisi, Francesca Lazzarini, Simona Del Quondam, Kashi Brunetti, Davide Cervia, Giorgio Prantera

**Affiliations:** 1 Department of Ecological and Biological Sciences (DEB), University of Tuscia, 01100 Viterbo, Italy; bongiorni@unitus.it (S.B.); francesca.lazzarini@studenti.unitus.it (F.L.); prantera@unitus.it (G.P.); 2 Department for Innovation in Biological, Agro-Food and Forest Systems (DIBAF), University of Tuscia, 01100 Viterbo, Italy; ecatalani@unitus.it (E.C.); simona.delquondam@unitus.it (S.D.Q.); kashi.brunetti@unitus.it (K.B.); 3 Bioinformatics Facility, European Brain Research Institute (EBRI) “Rita Levi-Montalcini”, 00161 Rome, Italy; i.arisi@ebri.it; 4 Institute of Translational Pharmacology (IFT), National Research Council (CNR), 00133 Rome, Italy

**Keywords:** amyloid β peptides, Alzheimer’s disease, brain neurodegeneration, Drosophila model system, Lisosan G, nutraceuticals, natural compounds

## Abstract

Alzheimer’s disease (AD) brains are histologically marked by the presence of intracellular and extracellular amyloid deposits, which characterize the onset of the disease pathogenesis. Increasing evidence suggests that certain nutrients exert a direct or indirect effect on amyloid β (Aβ)-peptide production and accumulation and, consequently, on AD pathogenesis. We exploited the fruit fly *Drosophila melanogaster* model of AD to evaluate in vivo the beneficial properties of Lisosan G, a fermented powder obtained from organic whole grains, on the intracellular Aβ-42 peptide accumulation and related pathological phenotypes of AD. Our data showed that the Lisosan G-enriched diet attenuates the production of neurotoxic Aβ peptides in fly brains and reduces neuronal apoptosis. Notably, Lisosan G exerted anti-oxidant effects, lowering brain levels of reactive oxygen species and enhancing mitochondrial activity. These aspects paralleled the increase in autophagy turnover and the inhibition of nucleolar stress. Our results give support to the use of the Drosophila model not only to investigate the molecular genetic bases of neurodegenerative disease but also to rapidly and reliably test the efficiency of potential therapeutic agents and diet regimens.

## 1. Introduction

Alzheimer’s disease (AD) stands as the most prevalent neurodegenerative disorder and form of dementia, characterized by cognitive decline typically occurring later in life [1]. Histologically, AD brains exhibit intracellular and extracellular amyloid deposits [2,3,4]. These amyloid plaques primarily consist of amyloid peptides Aβ-40 and Aβ-42, with the latter being the predominant amyloidogenic peptide, generated through differential proteolytic cleavage of the transmembrane receptor Amyloid Precursor Protein (APP). This cleavage is mediated by the β-site APP-cleaving enzyme (BACE) and the γ-secretase complex comprising Presenilin 1 and 2, Nicastrin, APH-1, and PEN-2 [5,6]. Excessive accumulation of amyloid β (Aβ) peptides is thought to be the initial event in the disease pathogenesis, inducing neuronal dysfunction and death [7,8].

The power of the fruit fly *Drosophila melanogaster* genetics to model neurodegenerative diseases provides novel insights into the pathogenic processes that occur in human brains [9,10,11,12]. Approximately 70% of genes responsible for human diseases have counterparts in the fruit fly [13]. Hence, it is not unexpected that many genes linked to AD pathogenesis have counterparts in Drosophila that display functional similarity. The Drosophila homolog of human APP, known as APP-like or APPL, possesses characteristic domains akin to vertebrate APP family members but lacks similarity at the C-terminal amyloidogenic Aβ-peptide sequence [14]. For this reason, despite the conservation and functional capabilities of γ-secretase complex components in Drosophila, which can accurately process both human APP and fly APPL [15,16,17,18], it was thought that no endogenous toxic Aβ-peptide could be produced in the fly [19,20,21]. However, a β-secretase-like enzyme was later identified in Drosophila, able to cleave human APP and also Drosophila APPL, producing an Aβ-peptide able to aggregate and induce neurodegeneration phenotypes in Drosophila [16]. Overall, the above data show that APP and APPL proteins and their processing pathways are evolutionarily conserved, as is the production of a neurotoxin Aβ-peptide.

To gain further insights into APP processing, Aβ-peptide production, and neuro-degeneration induction, transgenic flies that carry Gal4-driven [22] constructs co-expressing the 695 amino acid isoform of human APP and the human β-site APP-cleaving enzyme 1 (BACE1), were generated [15,23,24]. Human APP undergoes cleavage by human BACE1 followed by endogenous Drosophila γ-secretase, resulting in the generation of the Aβ-peptide in the brains of transgenic flies. The intracellular accumulation and aggregation of Aβ align with AD phenotypes [25].

The development of fruit fly models of AD provides an excellent tool to assay the effects of diet on pathological phenotypes associated with Aβ peptide accumulation [26]. During the last few years, increasing evidence has suggested that certain nutrients exert a direct or indirect effect on Aβ-peptide production and accumulation and, consequently, on AD pathogenesis. Specific nutrients show modulating effects on the inflammatory response and on the oxidative stress related to the disease, which eventually leads to the neurodegeneration observed in AD [27]. Drosophila melanogaster is a potent in vivo model for studying human neurodegenerative diseases that could be used parallel to traditional vertebrate systems [12,28]. Furthermore, Drosophila models are valuable for exploring pathophysiological alterations and drug discovery, including investigations on bioactive natural compounds [29,30,31,32]. In this respect, Drosophila is a robust and well-established genetic framework. It provides a significant advantage concerning low-cost animal husbandry management, with a short generation time and lifespan, which are suitable characteristics to test the biological effects of a nutraceutical molecule in vivo [33,34,35].

To support the use of in vivo alternatives to the traditional vertebrate models in the study of the potential activity of natural substances in AD, recently, an interesting set of experiments highlighted the remarkable anti-oxidant properties of plant edible flower extract [36,37]. Lisosan G is a fermented powder obtained from organic whole grains (*Triticum aestivum*). It is a crude mix containing proteins, lipids, glucids, polysaccharides, vitamins B1, B2, and B6, tocopherols, polyunsaturated fatty acids [38], and mineral elements such as magnesium, iron, zinc, copper, and selenium [38]. Recently, an active role of the main metabolite components of Lisosan G, namely gallic acid, 4-hydroxybenzoic acid, quercetin, and nicotinamide, was suggested in mice retina affected by glaucoma [39]. In addition, the protective effects of Lisosan G against apoptosis, autophagy impairment, and oxidative stress/inflammation through the involvement of anti-oxidant systems are well documented in different models, including nervous system components of Drosophila [40,41,42,43,44,45,46]. In the present work, we exploited the Drosophila model of AD to evaluate in vivo the beneficial properties of the oral administration of Lisosan G on the brain content of Aβ-42 peptide and their pathological effects. 

## 2. Materials and Methods

### 2.1. Chemicals 

The alimentary integrator Lisosan G, which is registered with the Italian Ministry of Health as a nutritional supplement, was obtained from Agrisan Company (Larciano, Pistoia, Italy). As previously detailed [46], Lisosan G is a powder obtained by the fermentation of the bran and germ of a grain (*Triticum aestivum*) that contains different major components, including proteins, lipids, glucids, polysaccharides, oligo-elements, vitamins, and fatty acid and appears particularly rich in anti-oxidant components such as phenolic components and alpha-lipoic acid.

Bovine serum albumin, normal goat serum, anti-nitrotyrosine primary antibody (#A21285), and Alexa Fluor secondary antibodies were purchased from ThermoFisher Scientific (Monza, Italy). Anti-cleaved caspase 3 primary antibody (#9664) and horseradish peroxidase (HRP)-conjugated secondary antibodies were purchased from Cell Signaling Technology (Danvers, MA, USA). Anti-Light-Chain 3 (LC3) (#ab128025), anti-p62/Sequestosome-1 (SQSTM1) (#P0067), and anti-tubulin (#T5168) were purchased from Merck Sigma-Aldrich (Darmstadt, Germany). The primer pairs for PCR analysis were purchased from Bio-Fab Research (Roma, Italy). Where not indicated, the other reagents were purchased from Merck Sigma-Aldrich.

### 2.2. Fly Strains and Crosses 

**Figure 1:**
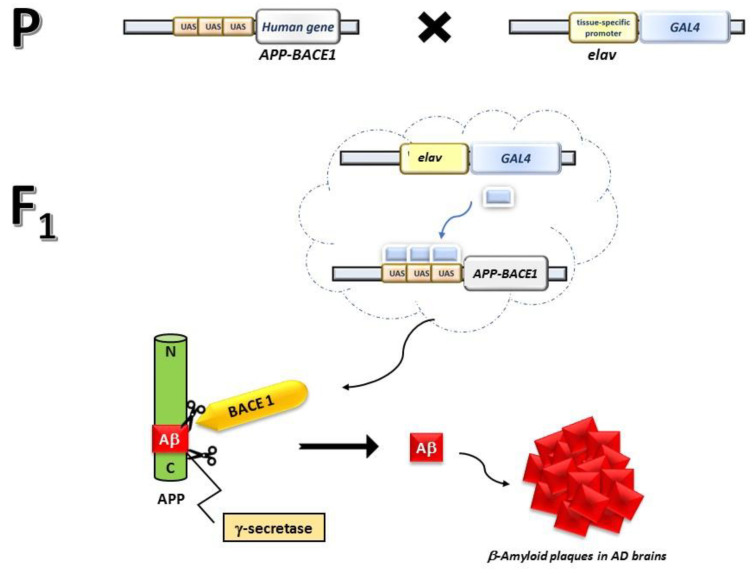
**Crossing to obtain progeny affected by AD in *D. melanogaster*.** The UAS/GAL4 system allows the expression of the human genes BACE1 (coding for the human β-secretase enzyme) and APP (coding for the human 695aa APP protein) throughout the central nervous system, resulting in the formation of Aβ plaques in the brain, a hallmark of AD. Specifically, by crossing transgenic parental lines (P) of Drosophila carrying the UAS sequence upstream the human genes APP and BACE1 with a pan-neuronal driver *elav-Gal4*, the F1 progeny generates the Aβ-42 expressing animals, which accumulates and forms amyloid plaques at the extraneuronal level.

Transgenic fly lines that express wild-type human Aβ-42 peptide (*UAS-Aβ42*) and a pan-neuronal *elav-Gal4* driver were obtained from Bloomington Drosophila Stock Center (Indiana University, Bloomington, IN, USA). The AD model flies were obtained by crossing three to five Gal4 virgin females to three Aβ-42 males. In the F_1_ progeny, AD animals were *UAS-Aβ42::ElavGAL4,* and they could be distinguished from healthy siblings based on their phenotypic characteristics (Figure 1). Healthy flies, recognized by the Tubby phenotype, do not contain the *UAS-Aβ42* third chromosome and hence did not reproduce the AD phenotype. In the nervous system of AD flies (phenotypically non-Tubby), Gal4 activated the expression of *APP* and *BACE1,* with the latter processing the former together with endogenous *D. melanogaster* γ-secretase, thus generating the toxic Aβ peptide.

### 2.3. Standard and Integrated Diet, Treatments

Flies were raised on a standard Drosophila cornmeal agar diet (STD) at 25 °C. Briefly, 1200 mL of diet contained 100 g cornmeal, 110 g glucose, 100 g yeast, and 8 g agar. As an anti-mold agent, 3 g ethyl 4-hydroxybenzoate, dissolved in 16 mL of absolute ethanol, was added to the diet. To prepare the experimental diet, Lisosan G was added to the STD and thoroughly mixed [46]. The generated AD model flies were allowed to feed on the diet supplemented with Lisosan G for the entire developmental period at a final concentration of 10 µg/mL [46]. Three crosses were set up as control, each on 10 mL of the STD; the other three crosses were set up on 10 mL of Lisosan G-supplemented diet. Parental strains (*UAS-Aβ42*) and the F_1_ healthy progeny were also used as a control in all the assays.

### 2.4. Tissue Preparation

Following published protocols [46,47,48,49,50], *D. melanogaster* heads were immersion-fixed overnight or for 48 h in 4% paraformaldehyde in 0.1 M Phosphate Buffer (PB) at 4 °C, transferred to 12% sucrose in PB, and stored at 4 °C for at least 24 h. Longitudinal sections (10 mm of thickness) were obtained by a cryostat, mounted onto positively charged slides, and stored at −20 °C until use. All the counts of the in situ immunolabeling experiments and the consequent statistical analyses were focused on the central brain area.

### 2.5. Aβ Immunohistochemistry—Congo-Red Staining

Histological brain sections (10 μm of thickness) were placed on slides and stained with an amyloid stain, Congo Red kit (Congo Red Stain Kit/Amyloid Stain; Abcam, Cambridge, UK), following the manufacturer’s protocol, with some slight modifications. Briefly, sections were quickly washed in PB; then, they were hydrated with distilled water. Slides were stained with 4–6 drops of Hematoxylin, incubated for 50–60 s, and rinsed with tap water. Slides were then stained with 4–6 drops of Bluing Reagent, incubated for 30 s, and rinsed in distilled water. Slides were dipped in 95% alcohol for 5 s and stained with Congo Red Solution for 20 min. Finally, slides were quickly dipped twice in 100% alcohol, dipped repeatedly (4 dips) in a clearing agent, and mounted in synthetic resin. 

### 2.6. Imaging and Quantification of Aβ Plaques

To quantify Aβ plaques in each brain, we analyzed brains from a minimum of 3 independent experiments; in particular, 12 brains from flies reared on STD and 15 brains from flies reared on the Lisosan G-supplemented diet. For image analysis, we used ImageJ software, available at: https://imagej.net/ij/ (accessed on 1 February 2024). Blinded quantification of Aβ plaques in histological preparations of Congo Red stained brain sections was performed by counting individual plaques in non-overlapping digital images captured using the 100× objective (Zeiss Axioskop 2 plus microscope; Carl Zeiss, Oberkochen, Germany). For the larger deposits of Aβ peptide, detectable as intense Congo Red staining, we also measured the area. Aβ plaques above 5 nm^2^ were considered as large.

### 2.7. Confocal Immunostaining

For immunostaining detection, longitudinal sections were washed in PB and then pre-incubated for 30 min at room temperature with 5% and 10% of normal goat serum in PB containing 0.5% Triton X-100. Pre-treated sections were incubated overnight at 4 °C with the following rabbit primary antibodies: anti-cleaved caspase 3 (1:500), anti-nitrotyrosine (1:100), anti-LC-3 (1:100), and anti-p62 (1:200) [51,52] in PB containing 0.5% Triton X-100. Following washes in PB, the sections were incubated in the appropriate Alexa Fluor secondary antibodies (1:200) in PB for 1.5 h at room temperature. Incubation in secondary antibody alone was performed as a negative control. 

Images were acquired by a Zeiss LSM 710 confocal microscope (Carl Zeiss). Blinded analysis of cleaved caspase 3 and nitrotyrosine immunostaining was carried out on the single images of each brain section. Each image was converted to grayscale and normalized to the background using Adobe Photoshop (Adobe Systems, Mountain View, CA, USA). Mean gray levels were then measured in the selected areas [53]. A minimum of 10 areas from at least 10 different flies collected in 3 independent experiments were analyzed for each experimental group. 

### 2.8. Protein Extraction and Western Blotting Analysis

Western blotting was carried out using protein extracts obtained from 15 fly heads per experimental group. Heads were homogenized in 20 μL of 6X sample buffer (comprising 0.125 M Tris-HCl pH 6.8, 2% SDS, 5% DTT, 10% Glycerol, Bromophenol Blue). The denatured protein samples were loaded onto a 12% polyacrylamide gel with a thickness of 0.75 mm for size-based separation. The running gel was prepared by mixing 30% acrylamide, 1.5 M Tris pH 8.8, 10% SDS, 10% APS and TEMED in a total volume of 8 mL distilled water. The stacking gel was prepared by mixing in distilled water, 30% acrylamide, 0.5 M Tris pH 6.8, 10% SDS, 10% APS and TEMED. Each well was loaded with 20 μL of protein sample. The gel-containing support was placed in the electrophoresis chamber with 1x TGS pH 8.3 and run for 1 h at a constant voltage of 120. 

For immunoblotting analysis [54,55], SDS–polyacrylamide gels were electroblotted onto a nitrocellulose membrane (Bio-Rad, Hercules, CA, USA) using transfer buffer (390 mM NaH_2_PO_4_H_2_O and 610 mM Na_2_HPO_4_H_2_O). The membranes were subsequently blocked with 5% low-fat dry milk and then probed with the rabbit anti-cleaved caspase 3 (1:500) and the mouse anti-tubulin (1:1000) antibodies. The incubation was conducted overnight at +4 °C. Following the primary antibody incubation, the membrane was washed with three 5-min rinses in the washing buffer and subsequently incubated with the appropriate HRP-conjugated secondary antibodies (1:5000) for an hour at room temperature. After the secondary antibody incubation, three additional 5-min rinses were performed. All blots were developed using the ECL Plus method (Amersham Biosciences/Cytiva, Freiburg, Germany) and signals were detected with the ChemiDoc scanning system (Bio-Rad) at multiple exposures.

### 2.9. Determination of Reactive Oxygen Species (ROS)

Determination of reactive oxygen species was performed as previously published with minor modifications [46]. Briefly, 100 fly heads per experimental group were weighted and then homogenized in 10 mM Tris buffer, pH 7. The homogenates were centrifuged at 1200 rpm for 5 min at 4 °C, and 100 µL of each supernatant were incubated in the presence of 5 µM 2′,7′-Dichlorofluorescin diacetate (DCFH-DA) at 37 °C for 60 min. Results were recorded at the end of the incubation at an excitation wavelength of 488 nm and an emission wavelength of 525 nm in a DTX 880 Multimode Detector (Beckman Coulter, Brea, CA, USA). 

### 2.10. 9 3-(4,5-Dimethylthiazol-2-yl)-2,5-Diphenyltetrazolium Bromide (MTT) Assay

The mitochondrial viability in the head homogenate of adult flies was evaluated as previously published with minor modifications [52,56,57]. Briefly, 100 heads per experimental group were weighted and manually homogenized in cold PB. The supernatants were collected after each consecutive centrifugation at 4 °C for 5 min at 1500 rpm. Mitochondrial activity was then evaluated using the MTT reduction method (0.5 mg/mL of final concentration) at the absorbance of 595 nm.

### 2.11. RNA Analysis and Gene Expression Assay

Frozen third instar larvae (n = 25) were homogenized in 600 mL of TRIzol solution (ThermoFisher Scientific). Total RNA was purified using the Direct-zol™ RNA MiniPrep Kits (Zymo Research, Irvine, CA, USA), following the manufacturer’s protocol. RNA integrity was checked on agarose gel 1%. RNA quantification was performed via Qubit RNA HS Assay Kit (ThermoFisher Scientific) using the Qubit 2.0 Fluorometer. mRNA was reverse transcribed with the RevertAid Reverse Transcriptase and the oligo(dT)18 primer in the first strand cDNA using the Thermo Scientific RevertAid First Strand cDNA Synthesis Kit (ThermoFisher Scientific) following the manufacturer’s instructions. The cDNA was synthesized in the thermocycler MJ Research and stored at −20 °C. Quantitative PCR was performed using the CFX96 Touch Real-Time PCR Detection System (Bio-Rad) with SYBR green dye (GoTaq^®^ qPCR Master Mix; Promega, Madison, WI, USA). The primer pairs are detailed in Table 1. Rpl32 has been used as a housekeeping gene for normalization using the 2^−ΔΔCT^ method.

### 2.12. Statistical Analysis

Generally, sample size calculation was conceptualized with a 5% alpha error, 80% power, and appropriate effect strength. Samples were only excluded from analyses due to technical problems, e.g., pipetting error, loss/spill of samples, or defects in materials/hardware. Statistical significance of raw data between the groups (completely randomized) in each experiment was evaluated using unpaired Student’s *t*/Mann–Whitney tests (single comparisons) or one-way ANOVA followed by the Tukey post-test (multiple comparisons). A *p*-value ≤ 0.05 was considered statistically significant. Data belonging to different experiments were represented and averaged in the same graph. R-Bionconductor [58], available at: https://www.r-project.org/ (accessed on 1 February 2024), and the GraphPad Prism 6 software package (GraphPad Software, San Diego, CA, USA) were used for the analyses. The results are expressed as means ± SEM of the indicated n values.

## 3. Results

To obtain *D. melanogaster* expressing the Aβ peptide in the brain, thus mimicking AD, transgenic Drosophila lines expressing the *human APP* and *BACE1* under UAS control were crossed to stocks expressing the pan-neuronal driver *elav-Gal4*. The generated AD model flies were allowed to feed either on STD or on the diet supplemented with Lisosan G for the entire developmental period at a final concentration of 10 µg/mL. This concentration was previously assessed as effective to counteract hyperglycemic injuries to Drosophila retinal neurons [46]. To minimize the effects of aging on the neurological phenotypes, the experiments were performed on 1–5 days-old adult animals unless otherwise indicated.

### 3.1. Lisosan G Diet Reduces the Number of Amyloid β Plaques in AD Brains

**Figure 2:**
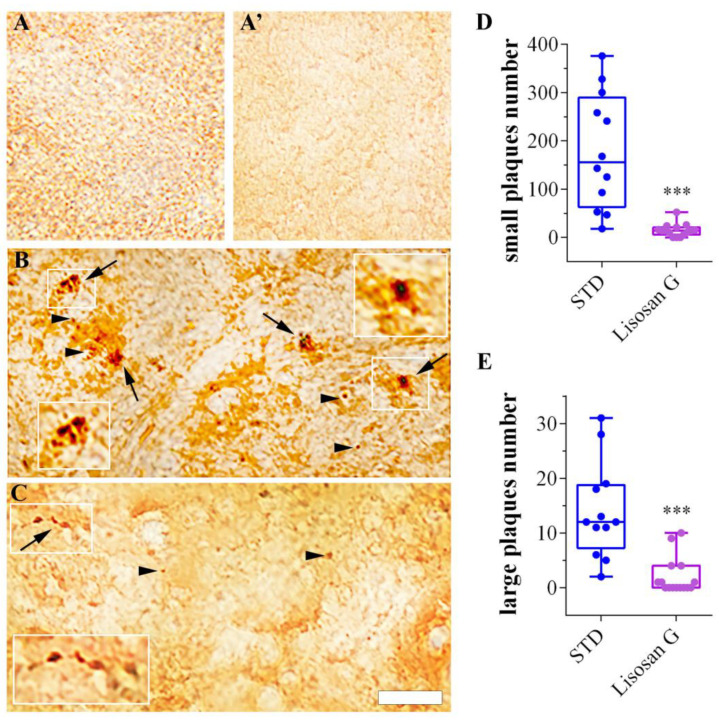
Effect of Lisosan G on Aβ plaques in AD brains of *D. melanogaster*. Longitudinal tissue sections from adult fly brains dyed with Congo Red staining to identify the Aβ plaques. (**A**,**A’**) Brain tissues from healthy individuals devoid of Aβ plaques grown on STD or food supplemented with Lisosan G, respectively. (**B**) In brain tissues from AD individuals, the Congo Red staining displays small clusters (arrowheads) and larger deposits of β-amyloid peptide (arrows). Inserts represent enlarged image details of the Aβ plaques. (**C**) Brain tissues from AD flies grown on food supplemented with Lisosan G, the Congo Red staining shows a remarkable recovery of the AD-related Aβ plaque phenotype. Scale bar: 10 μm. Quantitative measurements of small (**D**) and large (**E**) plaques number/brain in the brain of AD flies grown on STD or food supplemented with Lisosan G. *** *p* < 0.0001 vs. STD. Images and data are representative of n = 12–15 animals obtained from at least 3 independent experiments.

The amyloid structure of plaque-forming Aβ-peptide aggregates was identified through Congo Red staining [59] of tissue sections from adult fly brains, making this dye the gold standard for the histological visualization of amyloid in tissue sections. Brain tissues from healthy individuals grown on STD, with or without Lisosan G supplementation, were devoid of amyloid plaques (Figure 2A,A’), which instead characterized brain tissues from transgenic animals mimicking AD (Figure 2B). The Congo Red staining showed the presence of both numerous small clusters and a few larger deposits of Aβ peptide detectable as intense red staining in the brains of adult flies. The Congo Red staining on adult brain sections from AD flies grown on food supplemented with Lisosan G showed a remarkable recovery of the AD-related amyloid β plaque phenotype (Figure 2C), with a significant reduction in the β-amyloid peptide aggregates in all animals examined (Figure 2D,E). In total, we observed 2150 small plaques in the brains of 12 AD flies grown on standard food (mean: ca. 179 plaques/brain), as opposed to only 223 plaques in 15 AD flies grown on Lisosan G-supplemented food (mean: ca. 15 plaques/brain) (Figure 2D). As regards large plaques, we found 168 plaques in AD animals fed with STD (mean: 14 plaques/brain) that were significantly reduced in Lisosan-fed AD animals (total number: 30; mean: 2 plaques/brain) (Figure 2E). To note, the size of large plaques decreased as well in AD flies treated with Lisosan G when compared with animals grown on control food (Table 2).

### 3.2. Lisosan G Diet Reduces the Apoptosis Levels in AD Brains

**Figure 3:**
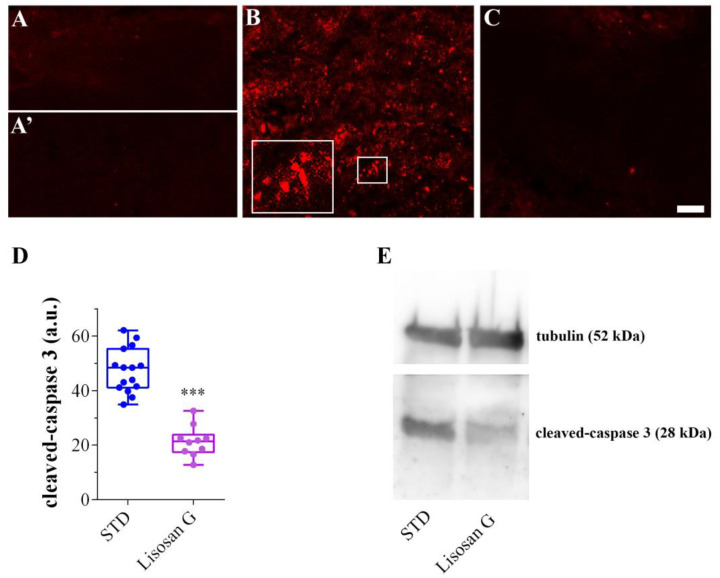
Effect of Lisosan G on the apoptosis levels of AD *D. melanogaster* brains. Confocal microscopy immunofluorescence imaging of cleaved (active) caspase 3 in longitudinal brain sections from healthy flies grown on STD (**A**) or food supplemented with Lisosan G (**A’**), AD flies grown on STD (**B**) or on food supplemented with Lisosan G (**C**). Inserts represent enlarged image details of the cleaved caspase-3 signals. Scale bar: 5 µm. (**D**) Quantitative analysis of cleaved caspase-3 immunofluorescence (a.u.: arbitrary units) in the brain of AD flies grown on STD or food supplemented with Lisosan G. *** *p* < 0.0001 vs. STD. Images and data are representative of n = 10–15 animals obtained from at least 3 independent experiments. (**E**) Western blot analysis of cleaved caspase 3 in adult brains from AD flies grown on STD and Lisosan G-supplemented diet. To avoid saturation, tubulin has been acquired at a shorter exposure than caspase 3. The proteins are thus displayed as separate blots cut at the relevant molecular weights. Image is representative of 3 independent experiments (n = 45 animals). Western blot original images are in the Supplementary Materials.

Drosophila AD adult brains were also characterized by intense apoptotic activity as evidenced by the presence of numerous cleaved (active) caspase-3 foci (Figure 3B), which were instead absent in non-AD brains from healthy flies grown on STD, with or without Lisosan G (Figure 3A,A’). Remarkably, the number and intensity of apoptotic foci were significantly reduced by ca. 65% when flies were grown on Lisosan G-supplemented food (Figure 3C,D). Accordingly, the immunoblotting analysis performed on protein extracts obtained from the heads of adult AD Drosophila confirmed a clear decrease in active caspase-3 in the brains of flies being administered with Lisosan G (Figure 3E).

### 3.3. Lisosan G Diet Reduces the Oxidation Levels of AD Brains

**Figure 4:**
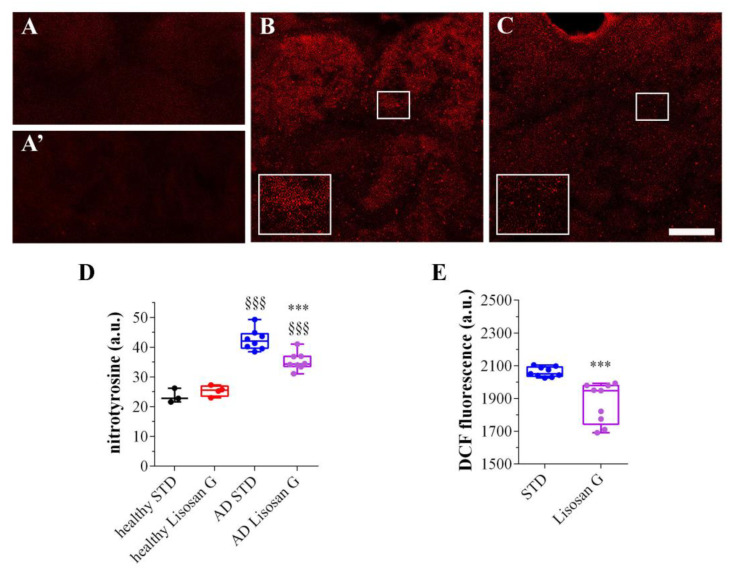
Effect of Lisosan G on the redox status of AD *D. melanogaster* brains. Confocal microscopy immunofluorescence imaging of nitrotyrosine in longitudinal brain sections from healthy flies grown on STD (**A**) or food supplemented with Lisosan G (**A’**) and AD flies grown on STD (**B**) or food supplemented with Lisosan G (**C**). Inserts represent enlarged image details of the nitrotyrosine signals. Scale bar: 20 µm. (**D**) Quantitative analysis of nitrotyrosine immunofluorescence (a.u.: arbitrary units). Images and data are representative of n = 5–8 animals obtained from at least 3 independent experiments. §§§ *p* < 0.0001 vs. healthy; *** *p* < 0.0001 vs. AD STD. (**E**) Measurements of ROS by DCF fluorescence intensity in heads of AD flies grown on STD or food supplemented with Lisosan G. Results are expressed as arbitrary units (a.u.). *** *p* < 0.0001 vs. STD. Data are representative of at least n = 300 animals obtained from 3 independent experiments run in triplicate.

A growing body of evidence has been accumulating in support of the beneficial role of polyphenols in preventing neurodegenerative diseases and relieving the symptoms of AD [60,61]. The Lisosan G’s ability to ameliorate the pathological symptoms in the Drosophila AD model can be ascribed to its polyphenol content, which was demonstrated to efficiently counteract oxidative stress [44]. In this respect, the redox status of AD flies was first verified by confocal immunostaining using an anti-nitrotyrosine antibody to detect peroxynitrite [49]. As shown in Figure 4A,A’,D, adult brains of healthy flies grown on STD, with or without Lisosan G, were nearly devoid of any peroxynitrite staining. On the contrary, intense nitrotyrosine immunostaining, formed by many punctuated signals, was clearly detected in all the areas of AD brains and was more intense when compared with animals grown on control food (Figure 4B–D). In AD flies fed with Lisosan G, instead, the increase in peroxynitrite labeling was significantly reduced by ca. 38%, although the oxidation levels did not reach those detected in healthy brains (Figure 4C,D).

The anti-oxidant effects of Lisosan G were then confirmed using the DCFH-DA probe. We found that DCF fluorescence in adult *D. melanogaster* AD heads was significantly decreased by Lisosan G, indicating lower levels of brain ROS (Figure 4E).

**Figure 5:**
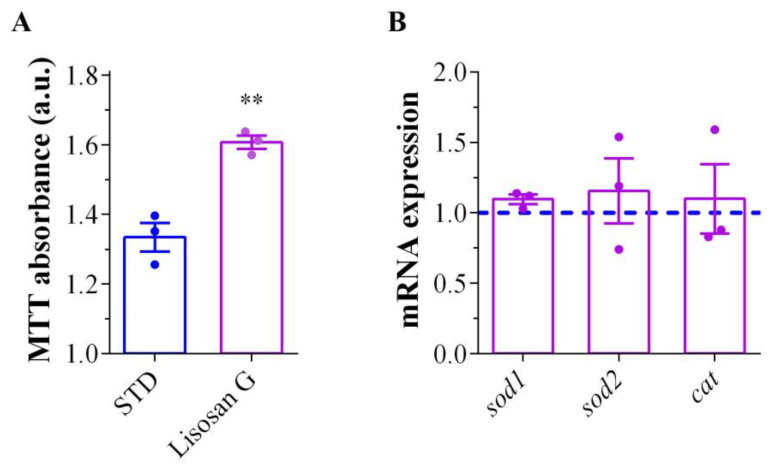
Mitochondrial activity and anti-oxidant enzyme levels in AD *D. melanogaster*. (**A**) Measurements of mitochondrial activity by MTT absorbance in brains of AD adult flies grown on STD or food supplemented with Lisosan G. Results are expressed as arbitrary units (a.u.). ** *p* < 0.001 vs. STD. Data are representative of n = 300 animals obtained from 3 independent experiments. (**B**) mRNA levels of sod1, sod2, and cat genes by qPCR in third instar larvae of AD flies grown on STD (blue dashed line) or food supplemented with Lisosan G (purple bars). Results are expressed as fold change of AD STD (blue dashed line). Data have been obtained from 3 independent experiments (n = 75 animals).

In support of the beneficial anti-oxidant effect of this treatment, we observed a significant enhancement in the MTT reductive ability in the brains of AD adult flies fed with Lisosan G in comparison to those under the STD condition (Figure 5A). These results prompted us to investigate the transcription differences of three enzymes involved in the cellular anti-oxidant response, namely superoxide dismutase (SOD) 1, SOD2, and catalase (CAT). However, similar mRNA levels were found in AD on STD or Lisosan G-supplemented medium (Figure 5B).

### 3.4. Lisosan G Diet Increases Autophagy Turnover of AD Brains

**Figure 6:**
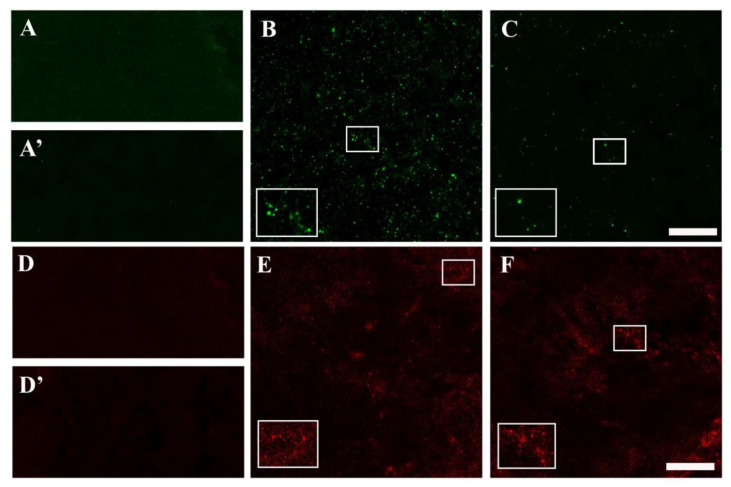
Effect of Lisosan G on the autophagy levels of AD *D. melanogaster* brains. Confocal microscopy immunofluorescence imaging of LC3 (**A**–**C**) and p62 (**D**–**F**) in longitudinal brain sections from healthy flies grown on STD (**A**,**D**) or food supplemented with Lisosan G (**A’**,**D’**) and AD flies grown on STD (**B**,**E**) or food supplemented with Lisosan G (**C**,**F**). Inserts represent enlarged image details of fluorescence signals. Scale bar: 20 μm. Images are representative of n = 5–8 animals obtained from at least 3 independent experiments.

Given the interplay between oxidative stress and the catabolic process autophagy, changes in autophagic function in Drosophila were detected by immunofluorescence analysis of the autophagic vacuole proteins LC3 and p62 [48,49]. Both LC3 and p62 staining was very faint in the adult brains of healthy flies grown on STD, with or without Lisosan G (Figure 6A,A’,D,D’). AD flies showed a large amount of LC3 clusters with intense fluorescent aggregates, while their presence was clearly reduced in the Lisosan G-treated group (Figure 6B,C). In contrast, punctate p62 immunostaining in untreated AD brains was similar to what was observed after Lisosan G administration (Figure 6E,F). 

### 3.5. Effect of Lisosan G on Nucleolar Stress

**Figure 7:**
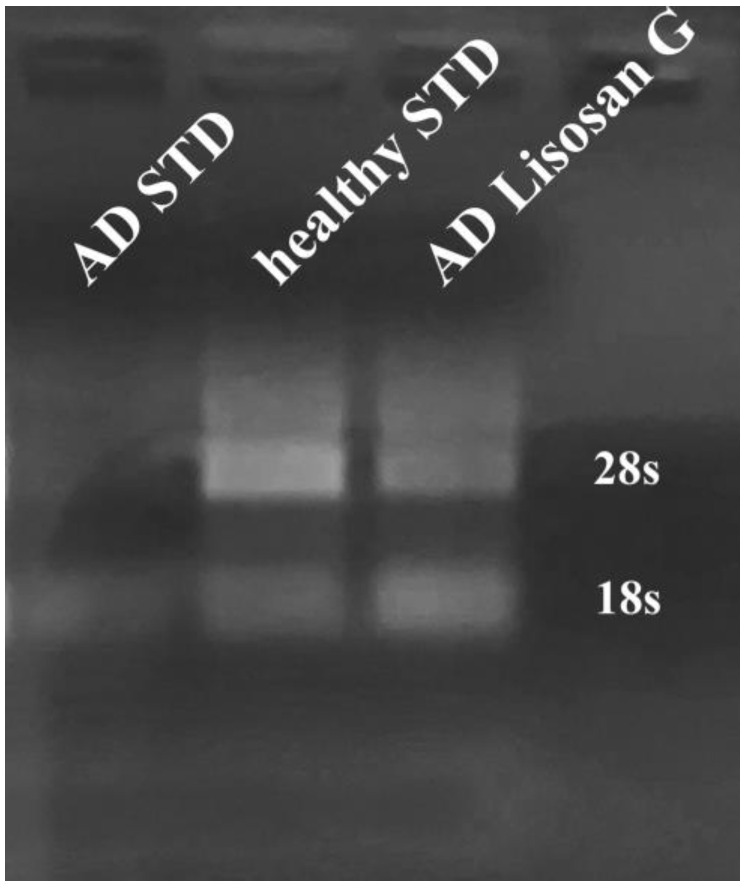
Effect of Lisosan G on nucleolar stress of *D. melanogaster*. Gel electrophoresis of RNA extracted from healthy (central lane) larvae showed the typical smear with the two broad bands corresponding to 18S and 28S RNAs. In AD larvae (left lane), the 28S rRNA band fully disappeared, whereas the 18S rRNA band became faint. In AD larvae grown on food supplemented with Lisosan G (right lane), the 18S and 28S bands were fully restored. Image is representative of 3 independent experiments (n = 75 animals).

Nucleolar stress is described as a cellular feature associated with AD in humans and mice and consists of a reduced expression and/or stability of the major ribosomal RNA species *18S* and *28S* in brain cells [62]. Gel electrophoresis of RNA extracted from healthy larvae showed the typical smear with the two broad bands corresponding to 18S and 28S RNAs (Figure 7, central lane). In AD larvae, the 28S rRNA band fully disappeared, whereas the 18S rRNA band became faint (Figure 7, left lane). Strikingly, in Lisosan G-fed AD larvae, the bulk RNA smear became again clearly visible and, in particular, the pattern of the two rRNA bands, notably that of 28S, was restored (Figure 7, right lane), even if the band intensity did not equal that of healthy brains.

## 4. Discussion

Using flies genetically engineered to mimic AD we tested the beneficial role of the natural product Lisosan G, commercialized as a nutritional supplement. Our results, summarized in Table 3, showed that the Lisosan G-enriched diet is capable of rescuing the AD typical phenotypes under analysis.

The distinctive tissue feature of AD is the accumulation of extracellular Aβ plaques in brains [6,63]. In the brains of transgenic adult Drosophila flies, co-expressing human *APP* and *BACE1*, we observed both large and small amyloid β plaques, which were absent in non-AD brains. The presence of these two typologies of plaques was already described in AD Drosophila, even though in a different nervous district, the retina, by [15], who observed a gender-biased distribution of them, with the larger plaques more evident in male retinas and the smaller ones limited to female retinas. Instead, our observations evidenced both large and small plaques in the same brain, irrespective of gender. Remarkably, Lisosan G treatment was able to strongly reduce the formation of amyloid β plaques, whether small or large. As it stands, we cannot discriminate whether this reduction is imputable to an inhibition of plaque formation or to an efficient disaggregation of already formed plaques. However, one can argue that small plaques came from the fragmentation of the large ones because of the Lisosan G action. Nevertheless, this is highly unlikely since the large-to-small plaque ratio was nearly identical in Lisosan G-treated vs. untreated AD flies (0.11 vs. 0.10). Moreover, if that hypothesis held, we should have observed an inverse relationship between the number of small and large plaques in a single Lisosan G-fed animal, but this is not the case.

Aβ plaques are also responsible for mitochondrial damage and dysfunction in AD [64]. As a consequence of mitochondrial impairment, AD brains are also characterized by a high rate of apoptosis [65,66,67,68], which is also a feature of the AD Drosophila model [69]. Wang and Davis [70] showed that neuron apoptosis in AD Drosophila brains is a consequence of Aβ-42-induced mitochondrial damage. Thus, the significant reduction in neuronal apoptosis following the Lisosan G diet is likely caused by the ability of this compound to rescue the mitochondrial damage in AD flies as a byproduct of the reduction in Aβ plaque number. In this respect, we found an increase in mitochondrial activity exerted by Lisosan G in the Drosophila AD brain.

Another archetypal AD-related phenotype is oxidative stress in neuronal cells [71]. In humans, mitochondrial damage appears in strict relationships to oxidative stress in AD brains, which is caused by mitochondrial dysfunctioning further enhanced by ROS accumulation [61,72,73]. In this scenario, it is fully coherent that Lisosan G can significantly reduce ROS levels and nitrotyrosine labeling in AD flies. Nitrotyrosine is the product of the action of the free radical oxidant peroxynitrite [74]. Nitrotyrosine represents an indicator of protein oxidation and is considered a reliable biomarker of oxidative stress in neurodegenerative diseases [75]. Moreover, nitrotyrosine was shown to mediate the Aβ peptide neurotoxicity [76]. Thus, these results clearly show that Lisosan G is also capable of relieving AD-related oxidative stress. In normal brains, oxidative stress triggers a cascade of anti-oxidant genes coding for enzymes acting as free radical scavengers, such as SOD1, SOD2, and CAT [77]. Literature data on the anti-oxidant gene expression in AD are controversial, being reported in different studies as a reduction, an increase, or invariance of anti-oxidant enzyme expression in the brains of affected humans. The discrepancies among the studies were ascribed to different disease stages and/or to the different sizes of the samples in different studies [78]. Our data indicate that Lisosan G did not affect SOD1, SOD2, and CAT expression levels, suggesting that its antioxidative action is exerted upstream of ROS formation. In this line, the increase in mitochondrial activity in AD brains implies a favorable influence of Lisosan G on mitochondrial functionality, indicating a beneficial effect on the main source of cellular ROS. Targeting both mitochondria and redox homeostasis emerges as a potential clinical option in different (neuro)degenerative diseases [79,80,81]. 

Additionally, Lisosan G is a nutraceutical powder enriched with a pool of bioactive substances, making it challenging to determine the specific pathways and identify the main genes through which it exerts its effects. Since it can be used as a nutraceutical approach with no harmful effects, Lisosan G deserves further exploration and appears to be a promising natural substance for potential use in AD conditions. Previous investigations into Lisosan G’s protective effects on Drosophila neurons after metabolic insults have indicated an interplay between apoptosis, autophagy, and ROS [46]. Similar results in flies were recently achieved with different natural compounds [52]. Additionally, mechanistic insights into Lisosan G’s action on regulating redox metabolites revealed its crucial role in determining the redox environment and interactions with free radicals [46]. For instance, Lisosan G increased the level of GSH, thus restoring the GSH/GSSG ratio and enhancing the anti-oxidant capacity of the glutathione anti-oxidant defense system. This may also occur in AD conditions. Of note, the increase in LC3 and p62 observed in AD brains of flies revealed that autophagy is somewhat impaired in our system with a high presence of accumulating autophagosomes awaiting lysosomal degradation. We found that Lisosan G exerted a positive effect on functional autophagy. Indeed, it lowered LC3-positive vacuoles, thus reactivating, at least in part, the autophagosome turnover of neurons. Autophagy, which is a primary intracellular mechanism for degrading aggregated proteins and damaged organelles, plays a crucial role in AD since oxidative stress may induce neuronal cell death/apoptosis via impairing autophagy of accumulated Aβ plaques [82]. 

Nucleolar stress is termed the function of the nucleolus as a cell stress sensor [83,84]. In neurodegenerative disease patients, including AD, nucleolar structure and functions become altered [62,85]. In this regard, Payão [86] demonstrated that 28S rRNA synthesis is reduced in the blood of AD patients as compared to age-matched healthy controls. This downregulation seems to result from rDNA promoter hypermethylation [87]. In line with the above observations, impairment of protein synthesis was found to represent an early event of AD pathogenesis [88]. In *D. melanogaster*, nucleolar stress was demonstrated in wild-type animals following ribosome biogenesis inhibition [89]. Our observation that a strong reduction in 18S and 28S rRNA is already apparent at the 3rd instar larval stage of transgenic *D. melanogaster* mimicking AD compellingly supports the notion that nucleolar stress represents an early diagnostic phenotype of AD pathology. On the other hand, the above observations further confirm that the AD Drosophila model recapitulates most of the human AD phenotypes. It is, thus, very interesting that Lisosan G feeding can also rescue this early AD pathological phenotype. This role of Lisosan G is conceivably related to the reduction in oxidative stress that has been indicated as responsible for nucleolar stress [90]. Also, impaired autophagy and defective mitochondria have been linked with nucleolar stress in AD [91].

## 5. Conclusions

In Drosophila, nutrient absorption takes place through the different gut sections, foregut, anterior midgut, middle midgut, and posterior midgut, like in humans [92]. Thus, flies provide the opportunity for rapid testing of new orally available therapeutic strategies. In this respect, the presented data demonstrate that the Lisosan G-enriched diet prevents and/or induces reversion of intraneuronal Aβ-peptide accumulation in the Drosophila model of AD. It also rescues other AD-related phenotypes and pathological mechanisms in the brain, namely apoptosis, oxidative and nucleolar stress, and autophagy impairment. Mechanistically, the beneficial effects of Lisosan G likely depend on Lisosan G-induced restoration of autophagy turnover/redox status and mitochondrial activity, which exert a key role against neuronal apoptosis and nucleolar stress. The promising data using the oral administration with Lisosan G against fly AD endorse the nutraceutical approach as a modern line of defense against AD neurodegeneration. However, they require further in-depth investigation and, potentially, comparison with other pharmacological/natural substances. The possibility of synergistic/additive beneficial effects of Lisosan G with other compounds could also pave the way for testing novel nutraceutical strategies in AD. 

*D. melanogaster* represents an acknowledged model system to investigate in vivo the genetic and molecular mechanisms underlying neurodegenerative human diseases, including AD, opening new avenues to the diagnosis and prognosis of such invalidating syndromes [12,21,26,93,94]. The ease of genetic modifications and treatments in flies also allows for early proof-of-principle studies of therapeutic approaches and drug screening. However, insects are evolutionarily and functionally distant from mammals and studies in flies have to be carefully analyzed before translating them to human pathophysiology. Although success rates of AD drugs in clinical trials after testing in vertebrate models have been disappointing, data obtained in Drosophila should be further verified in other organisms of increasing biological complexity in order to better model the disease for accurate use in preclinical studies.

As a whole, our results give further, strong support to the use of the Drosophila model not only to investigate the molecular genetic bases of neurodegenerative disease but also to rapidly and reliably test/screen the efficiency of potential therapeutic agents and diet regimens.

## Figures and Tables

**Table 1:** Primer pairs designed for qPCR analysis.

Gene Name	FlyBase ID	Primer Sequence *	Amplicon Size
*sod1*	FBgn0003462	F: 5′-ACCGACTCCAAGATTACGCTC-3′ R: 5′-CAGTGGCCGACATCGGAATA-3′	197 bp
*sod2*	FBgn0010213	F: 5′-AATCTAAATGCCGCCGAGGA-3′ R: 5′-CTCTTCCACTGCGACTCGAT-3′	197 bp
*cat*	FBgn0000261	F: 5′-CTATGGCTCGCACACCTTCA-3′ R: 5′-TCGTCCAACTGGGGAACTTG-3′	194 bp
*rpl32*	FBgn0002626	F: 5′-GACCATCCGCCCAGCATAC-3′ R: 5′-CGGCGACGCACTCTGTT-3′	138 bp

* F: forward, R: reverse.

**Table 2:** Area of Aβ large plaques in AD *D. melanogaster* brains.

Treatment	Mean Area (nm^2^)
STD	19.65 ± 1.83
Lisosan G-supplemented food	11.32 ± 2.03 ***

*** *p* < 0.0001 vs. STD.

**Table 3:** An overview of the effects of Lisosan G on different AD Drosophila phenotypes.

	Healthy	AD	AD + Lisosan G
Aβ plaques	−	+	−
Apoptosis	−	+	−
Oxidative stress	−	+	−
Autophagy	●	‡‡	●
18S and 28S rDNA stability	+	−	+

“−” indicates absence/low levels and “+” presence/high levels of the given phenotype. “●” indicates functional autophagy and “‡‡” impaired autophagy.

## Data Availability

The data analyzed during this study are included in this published article. Additional supporting data are available from the corresponding authors upon reasonable request.

## Supplementary Materials

The following supporting information can be downloaded at: https://www.mdpi.com/article/10.3390/biom14070855/s1, Figure S1: Figure 3E Western blot original images.
