# Supplementary figures and images for: Pathological Defects in a Drosophila Model of Alzheimer’s Disease and Beneficial Effects of the Natural Product Lisosan G
Source: Biomolecules. 2024 Jul 15;14(7):855. doi: 10.3390/biom14070855 (PMC11274821; doi:10.3390/biom14070855)

original blot

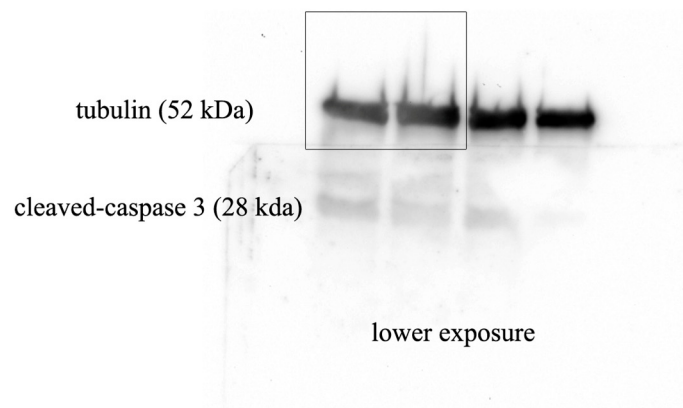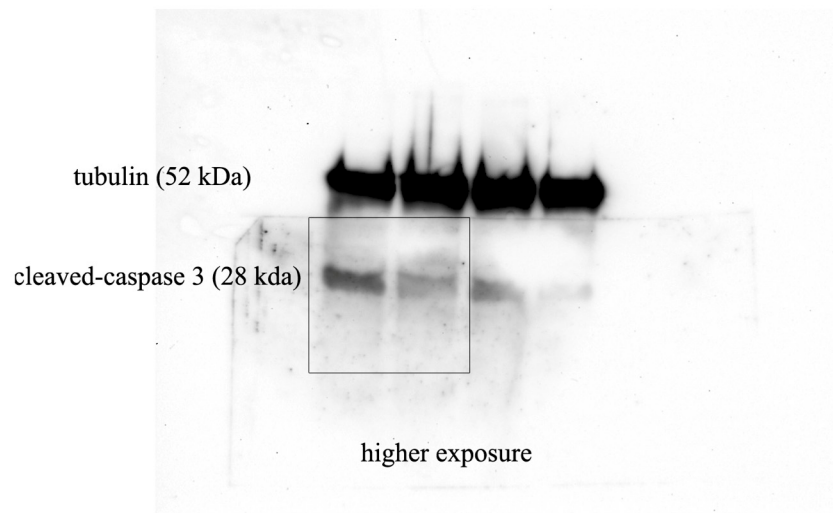

**Figure S1.** Figure 3E Western blot original images.

Supplement: Supplementary file 1 [file biomolecules-14-00855-s001.zip › biomolecules-3053973-Supplementary.pdf]
